# Safety and effectiveness of outpatient laparoscopic cholecystectomy in a teaching hospital: a prospective study of 110 consecutive patients

**DOI:** 10.1186/1756-0500-3-207

**Published:** 2010-07-22

**Authors:** Athanasios Marinis, Emmanouil Stamatakis, Athanasia Tsaroucha, Nikolaos Dafnios, Georgios Anastasopoulos, Georgios Polymeneas, Theodosios Theodosopoulos

**Affiliations:** 1 Second Department of Surgery Aretaieion University Hospital, Faculty of Medicine, National and Kapodistrian University of Athens, 76 Vassilisis Sofia's Ave, 11528, Athens, Greece; 2 First Department of Anesthesiology, Aretaieion University Hospital, Faculty of Medicine, National and Kapodistrian University of Athens, 76 Vassilisis Sofia's Ave, 11528, Athens, Greece

## Abstract

**Background:**

The aim of this study was to evaluate the safety and efficacy of outpatient laparoscopic cholecystectomy (OLC) in a day surgery unit in a teaching hospital. OLC was offered to patients with symptomatic cholelithiasis who met the following established inclusion criteria: ASA (American Society of Anesthesiology) physical status classification class I and II; age: 18 - 70 years; body mass index (BMI) < 30 kg/m^2^; patient acceptance and cooperation (informed consent); presence of a responsible adult to accompany the patient to his residency; patient residency in Athens. The primary study end-point was to evaluate success rates (patient discharge on the day of surgery), postoperative outcome (complications, re-admissions, morbidity and mortality) and patient satisfaction. A secondary endpoint was to evaluate its safe performance under appropriate supervision by higher surgical trainees (HSTs).

**Findings:**

110 consecutive patients, predominantly female (71%) and ASA I (89%) with a mean age 40.6 ± 8.1 years underwent an OLC. Surgery was performed by a HST in 90 patients (81.8%). A mean postoperative pain score 3.3 (range 0-6) occurred in the majority of patients and no patient presented postoperative nausea or vomiting. Discharge on the day of surgery occurred in 95 cases (86%), while an overnight admission was required for 15 patients (14%). Re-admission following hospital discharge was necessary for 2 patients (1.8%) on day 2, due to persistent pain in the umbilical trocar site. The overall rate of major (trocar site bleeding) and minor morbidity was 15.5% (17 patients). At 1 week follow-up, 94 patients (85%) were satisfied with their experience undergoing OLC, with no difference between grades of operating surgeons.

**Conclusions:**

This study confirmed that OLC is clinical effective and can be performed safely in a teaching hospital by supervised HSTs.

## Background

Laparoscopic cholecystectomy (LC) has become in recent years the standard approach for managing symptomatic cholelithiasis and is thus accepted as the "gold standard" surgical technique [1,2]. With improvements in anesthesia and perioperative care LC was attempted as an outpatient cost-effective procedure, despite several concerns about patient safety which initially halted its worldwide acceptance [3-8].

Besides clinical effectiveness and cost saving, LC is associated with several complications, such as bleeding and bile duct injuries, which can occur in open cholecystectomy as well, are usually detected intraoperatively and lengthen hospital stay [3,9]. Similarly, postoperative nausea and vomiting (PONV) as well as pain are regarded as key factors influencing same day discharge and several methods have been implicated to reduce both of them [10-14].

In Greece, outpatient cholecystectomy is gradually gaining acceptance from surgeons and patients. The aim of this study was to evaluate the outcome of outpatient LC (OLC) in a teaching university hospital, confirming the safety of performing this procedure by higher surgical trainees (HSTs).

## Methods

### Patient characteristics

This clinical study was performed after the approval of the ethics committee of our hospital. During a 3-year period (Jan 2005 - Dec 2007), patients undergoing elective outpatient laparoscopic cholecystectomy (OLC) in our purpose-built (i.e. for the purpose of day surgery cases) Day Surgery Unit at Aretaieion University Hospital were prospectively studied. This unit is run by dedicated medical, nursing and administrative staff and is open from 07:00 a.m. until 21:00 p.m.

OLC was offered to patients with ultrasound-documented symptomatic cholelithiasis who met the following established inclusion criteria: ASA (American Society of Anesthesiology) physical status classification class I and II; age: 18 - 70 years; body mass index (BMI) < 30 kg/m^2^; patient acceptance and cooperation (informed consent); presence of a responsible adult to accompany the patient to his residency and staying with him overnight; patient residency in Athens. Prior upper abdominal surgery was not considered as an exclusion criterion. Patients with jaundice or dilated bile ducts on ultrasonography were excluded and offered further diagnostic work-up. Delayed surgical treatment of acute cholecystitis was considered as an exclusion criterion, due to the possibility of a higher conversion rate. After selection, patients underwent clinical examination by the anesthesiologist and had an electrocardiogram which was evaluated by the cardiologist of the department and blood was drawn for determining total blood count and liver function tests.

Surgery was performed by consultant surgeons and supervised HSTs, which are residents in postgraduate years 5 and 6. All OLCs were scheduled on a morning list in order to permit enough time for patient recovery prior to day surgery unit closure in the afternoon. All patients received standard anti-DVT prophylaxis (preoperatively administered elastic stockings, intraoperative pneumatic compression).

The following discharge criteria were used for all patients: awake, oriented, mobilized and tolerating oral fluids; passage of urine; no postoperative pain, nausea or vomiting; stable general condition for at least 120 min evaluated by the consultant surgeon and anesthesiologist.

Follow-up (questioning for post-operative pain or discomfort, nausea or vomiting, overall satisfaction, return to work) was done by telephone contact the first day after surgery, by clinical examination on an outpatient basis one week after operation and by telephone contact one month postoperatively.

### Anesthetic technique

Patients did not receive any premedication. In the operating room, patients were attached to a bispectral index (BIS) monitor for evaluation of depth of anesthesia and to a standard hemodynamic monitoring (electrocardiogram, oxygen saturation, heart rate, and non-invasive blood pressure). Intraoperatively, end tidal carbon dioxide and sevoflurane concentration were also monitored.

After inserting a peripheral venous catheter (18G) all patients received 1 mg midazolam, 50 mg ranitidine and 10 mg metoclopramide. After preoxygenation for 3 min, anesthesia was induced with fentanyl 3 μ g/kg, propofol 2.5 mg/kg followed by rocuronium 0.6 mg/kg to facilitate tracheal intubation and to provide muscle relaxation. To maintain anesthesia, we administered sevoflurane in 50% oxygen to air mixture. The inspired sevoflurane concentration was titrated to maintain a BIS value between 40 and 50. Prior to extubation (20-30 min) we intravenously administered paracetamol 1.2 g, parecoxib 40 mg, and ondasetron 4 mg.

Postoperative pain was controlled by a multimodal protocol: local infiltration of trocar sites with ropivacaine, short acting opioids, cyclo-oxygenase-2 inhibitors and paracetamol. Postoperative nausea and vomiting was prevented by the combined administration of metoclopramide and ondansetron. Both parameters were assessed with a 0-10 visual analogue scale (VAS): 0-4 (mild pain or nausea well tolerated), 5-7 (moderate pain or nausea needing antiemetics) and 8-10 (severe pain or vomiting).

### Operative technique

Pre-incisional infiltration of all trocar sites with 4 ml ropivacaine 0.375% was initially performed. Pneumoperitoneum of 12 mmHg was established via a 10 mm trocar inserted through a horizontal infraumbilical incision using the open (Hasson) approach. Additonal trocars were placed according to the North american four-trocar technique used in our department: a 10 mm subxiphoid trocar, a 5 mm trocar in the right midclavicular line subcostally and, finally, a 5 mm trocar approximately in the anterior axillary line subcostally. After recognizing the anatomic structures of the triangle of Callot (using the "critical view of safety" technique) endoclips were placed on the cystic artery and cystic duct, ligating them. A typical cholecystectomy was then performed. After ensuring adequate hemostasis and according to the surgeon's decision a passive drain was placed adjacent to the gallbladder fossa and remained for 3-4 hours. The drain was removed, unless a bile leak or active hemorrhage was suspected. No intraoperative cholangiography was routinely performed. Finally, antibiotics were not routinely administered during the perioperative period.

### Study end-points

The aim of this study was primary to evaluate success rates (patient discharge on the day of surgery), postoperative outcome (complications, readmissions, morbidity and mortality) and patient overall satisfaction for medical management, staff manners and hospital facilities (on a visual analogue scale: excellent 8-10; very good 6-8; good 3-5; bad 0-2) regarding OLC. A secondary end-point was to evaluate surgical training and more specifically the safety of performance of OLC by HSTs.

### Statistical analysis

Continuous data are expressed as mean ± standard deviation. Comparison of continuous data was performed with the two-sample t-test, while comparison of categorical data was performed using the chi-square test. A level = 0.05 of statistical significance was used. Analysis was performed using the statistical package Minitab (version 14, Pennsylvania, USA).

## Results

110 consecutive patients fulfilling the aforementioned inclusion criteria underwent an OLC in Aretaieion University Hospital's Day Surgery Unit. The patients were predominantly female (71%) and the mean age was 40.6 ± 8.1 years. The majority of patients were ASA I (89%) and the remainder were ASA II. Eleven patients had previously undergone upper abdominal surgery (3 liver resections for hydatid disease, 3 Billroth II gastrectomies, 2 total gastrectomies and 3 closures of duodenal perforation).

Surgery was performed by supervised HSTs in 90 patients (81.8%). The duration of the operation ranged from 25 to 65 min, with a mean overall (both HST and consultants) operating time of 43.8 ± 8.2 min. Mean operating times for consultants were significantly less (35.5 ± 6.38) than those for HSTs (45.76 ± 7.29), while overnight stay, postoperative nausea and vomiting (PONV), pain, readmission and morbidity rates did not differ significantly (Table 1). Both groups of patients were comparable regarding demographic data.

**Table 1:** Comparison of outcome of outpatient laparoscopic cholecystectomy (OLC) performed by consultant surgeons and higher surgical trainees (HSTs)

	Consultants (n = 20)	HSTs (n = 90)	*p *value
Operating time (min)	35.5 ± 6.38 *	45.76 ± 7.29 *	***< 0.001 ***†

PONV	0	0	-

Minor Postoperative pain (patients)	15 (75%)	80 (88.9%)	0.102 ‡

Admitted patients	2 (10%)	13 (14.4%)	0.6 ‡

Satisfied patients (at 1 month folow-up)	18 (90%)	82 (91.1%)	0.876 ‡

Re-admission	1	1	-

Overall Morbidity	3 (15%)	13 (14.4%)	0.949 ‡

* mean ± SD

† t-test

‡ chi-square test.

Conversion to open cholecystectomy was not necessary for any patient. Drains were selectively inserted in 60 patients (54.5%), according to the attending surgeon's judgment.

A mean postoperative pain score 3.3 (according to the VAS scale) occurred in the majority of patients and requirements for paracetamol were minimal. No patient presented nausea or vomiting.

Discharge on the day of surgery occurred in 95 cases (86%), while an overnight admission was required for 15 patients (14%). Reasons for overnight stay included unavailability (due to family or other social problems) of a responsible adult in 5 patients, postoperative anxiety and discomfort in another 5, delay of the operating room schedule in 4 patients and bleeding from a trocar incision in one case. The last patient was a 30-year-old female patient admitted due to ongoing bleeding from the right hypochondrial trocar site with a concomitant decrease of the hematocrit. Re-laparoscopy revealed the bleeding site and hemostasis was successfully achieved. The patient was finally discharged 48 hours after initial admission.

Re-admission following hospital discharge was necessary for 2 patients (2.1%) on day 2, due to persistent pain in the umbilical trocar site. A subcutaneous hematoma was diagnosed and percutaneously drained. In the remaining patients minor complaints included serum discharge from the umbilical trocar site in 3 patients, shoulder tip pain in 4 patients and abnormal liver function tests in 7 female patients one week after discharge, which subsided gradually within 3 weeks. The overall rate of major (trocar site bleeding) and minor morbidity was 15.5% (17 patients). No postoperative bile leaks or retained common bile duct stones were noted.

At 1 week follow-up, 94 patients (85%) were satisfied (VAS score > 6) with their experience undergoing OLC, with this remaining the same 1 month postoperatively. There was no significant difference between grade of operating surgeon and patient satisfaction (Table 1).

## Discussion

Improvements in anesthetic and surgical techniques have prompted surgeons to perform LC as an elective outpatient procedure. Recent systematic reviews and metanalyses have shown the safety and effectiveness of this method in selected patients, with reduced cost and high level of patient satisfaction [15-18].

Current available literature strongly suggests implementation of standard criteria for selection of patients for OLC. Fitness for surgery (ASA classes I and II), proximity to the hospital and the availability of a responsible adult are considered effective criteria for selecting patients, increasing success rates of OLC. In our study, using these criteria with the addition of age (18-70 years) and BMI (< 30 kg/m2) was associated with a success discharge on the day of surgery for 86% of our cases. However, less restrictive criteria (age > 70 yrs or any BMI) currently seem to have no or little effect on the success rates of OLC and are gradually implemented in our daily practice as well.

Serious complications after LC include bleeding and bile duct injuries. In our series, only one patient developed postoperative bleeding, which was successfully controlled during re-laparoscopy. No mortality was reported as well. A readmission rate of 1.8% for our patients is in accordance to that (2%) reported in several studies [14,19,20].

Postoperative nausea and vomiting is considered to be a major concern in cases of OLC, studied thoroughly in several randomized controlled studies, without however any agreement or specific consensus in premedications and anesthetic drug regimens [21-24]. PONV associated with ambulatory surgery accounts for 0.1 - 0.2% of unanticipated admissions [25]. Interestingly, in our study, no patient suffered from PONV, a major factor for early discharge and increased success of OLC. This probably was achieved due to the anesthetic technique used: administration of propofol for induction, avoidance of nitrous oxide, prophylactic intravenous administration of 4 mg ondasetron at the end of the procedure and adequate patient hydration.

In a similar manner, postoperative pain may increase hospital stay in the ambulatory setting [26]. Various drug regimens and other methods have been reported and proposed to prevent and reduce postoperative pain, which is a significant factor for early patient mobilization and increased satisfaction for the outpatient procedure [27-29]. Pre-incisional infiltration of portal sites as well as intraperitoneal infusion of local anesthetics, pethidine and normal saline have been reported as effective and reasonable options for pain control [4,6,10-12,30,31]. In our study, postoperative pain was minimal due to effective drug protocols and pre-incisional infiltration of ropivacaine in trocar sites.

Overall patient satisfaction reached 85% in our series and remains one of the most significant components of success of OLC. According to the literature, performance of LC by supervised HSTs is considered safe and does not affect patient outcome [32]. In our study, this issue was confirmed, despite any differences in the homogeneity (the "difficult" cases with previous upper GI surgery were mostly performed by consultants) and the size of both groups (HSTs vs. consultants).

## Conclusions

This study confirmed the recently appreciated advantages of outpatient LC concerning patient safety and clinical effectiveness. In addition, the performance of OLC by trainees has no negative implications on success rates of the procedure. Careful patient selection, standardized anesthetic protocols, advanced surgical technique and appropriate perioperative care in purpose built day surgical units are necessary prerequisites for the successful implementation of outpatient laparoscopic cholecystectomy in every country.

## Competing interests

The authors declare that they have no competing interests.

## Authors' contributions

AM, ES, AT and GA contributed significantly in conception, design and analysis and interpretation of data; AM and ES drafted the manuscript; AM, ES, ND, GP, TT critically revised the manuscript; AM, ES and TT gave final approval for publishing. All authors read and approved the final manuscript.
